# Leigh syndrome in a patient with a novel *C12orf65* pathogenic variant: case report and literature review

**DOI:** 10.1590/1678-4685-GMB-2018-0271

**Published:** 2020-05-29

**Authors:** Eduardo Perrone, Thiago R. Cavole, Manuella G. Oliveira, Luiza do A. Virmond, Marina de França B. Silva, Maria de Fatima F. Soares, Simone Brasil de O. Iglesias, Ariane Falconi, Juliana S. Silva, Viviane Nakano, Maria Fernanda Milanezi, Carmen Silvia C. Mendes, Marco Antonio Curiati, Cecília Micheletti

**Affiliations:** 1 Universidade Federal de São Paulo, Departamento de Genética Médica, São Paulo, SP, Brazil.; 2 Universidade Federal de São Paulo, Departamento de Radiologia, São Paulo, SP, Brazil.; 3 Universidade Federal de São Paulo, Departamento de Pediatria, São Paulo, SP, Brazil.; 4 GeneOne, Dasa, São Paulo, SP, Brazil.

**Keywords:** C12orf65, Leigh syndrome, COXPD7, mitochondrial, oxidative phosphorylation

## Abstract

Leigh syndrome is an early onset progressive disorder caused by defects in mitochondrial oxidative phosphorylation. Pathogenic variants in nuclear and mitochondrial genes are associated with the syndrome. Homozygous pathogenic variants in the *C12orf65* gene impair the mitochondrial oxidative phosphorylation system. We describe a new case of Leigh syndrome caused by a novel pathogenic variant of the *C12orf65* gene resulting in the lack of the Gly-Gly-Gln (GGQ) domain in the predicted protein, and review clinical and molecular data from previously reported patients. Our study supports that the phenotype caused by *C12orf65* gene variants is heterogeneous and varies from spastic paraparesis to Leigh syndrome. Loss-of-function variants are more likely to cause the disease, and variants affecting the GGQ domain tend to be associated with more severe phenotypes, reinforcing a possible genotype-phenotype correlation.

Leigh syndrome is an early-onset progressive neurodegenerative disorder associated with defects of mitochondrial oxidative phosphorylation and is the most common distinct phenotype among oxidative phosphorylation disorders in children. The syndrome affects 1 in 32,000 - 40,000 newborn children (Rahman *et al.*, 1996; Darin *et al.*, 2001), but higher incidences up to 1 in 2000 have been reported due to founder mutations in the Faroe Islands and in an isolated population near Quebec, Canada (Carrozzo *et al.*, 2007; Ruhoy *et al.*, 2014). The clinical criteria for the diagnosis include progressive neurological disease with motor and intellectual developmental delay, signs, and symptoms of brain stem and/or basal ganglia disease, raised lactate levels in blood and/or cerebrospinal fluid, and characteristic neuroimaging and/or postmortem neuropathological changes (Baertling *et al.*, 2014). The brain changes are almost identical in all patients, despite substantial clinical and genetic heterogeneity. The onset of symptoms is considered typical between 3 and 12 months of age (Thorburn *et al.*, 2003). The prognosis of Leigh syndrome is generally poor, with rapid deterioration of cognitive and motor functions resulting in death within months or a few years (Sofou *et al.*, 2014). This syndrome is genetically highly heterogeneous as evidenced by the causal association with variants in more than 75 mitochondrial or nuclear genes (Lake *et al.*, 2016).

The nuclear *C12orf65* gene at chromosome 12q24.31 comprises three exons, with 501 base pairs in its protein-coding exons 2 and 3. It encodes a soluble mitochondrial matrix protein related to the family of class I peptide chain release factors (RFs), which interacts with the large ribosomal subunit to release the polypeptide chain from the P-site-bound peptidyl-tRNA (Huynen *et al.*, 2012; Kogure *et al.*, 2012).

The C12orf65 protein consists of 166 amino acids that include an RF-1 domain (residues 53-146) and a GGQ motif (residues 71-73). This protein does not coprecipitate with mitochondrial ribosomes (Richter *et al.*, 2010) and has no peptidyl-tRNA hydrolase activity (Antonicka *et al.*, 2010).

Pathogenic variants in the *C12orf65* gene impair the mitochondrial oxidative phosphorylation system and are causative of combined oxidative phosphorylation deficiency 7 (COXPD7). To date, at least 11 pathogenic variants in this gene have been reported (Antonicka *et al.*, 2010; Shimazaki *et al.*, 2012; Buchert *et al.*, 2013; Heidary *et al.*, 2014; Spiegel *et al.*, 2014; Tucci *et al.*, 2014). Mutations were first described in two unrelated pedigrees affected by Leigh syndrome (LS; MIM #256000) and Leigh-like syndrome (Antonicka *et al.*, 2010).

The phenotypes associated with *C12orf65* variants may be classified into three groups: mild, intermediate and severe, distinguished by disease severity, mortality, and the lack or presence intellectual disability (Spiegel *et al.*, 2014). Here we describe a 2-year-old female patient with Leigh syndrome and a novel frameshift deletion pathogenic variant of the *C12orf65* gene. We also review clinical and molecular aspects of the clinical phenotype caused by *C12orf65* variants.

The 2-year-old girl was admitted to our hospital presenting with respiratory failure and sleepiness. Physical examination showed global hypotonia, developmental delay, and no dysmorphisms. She was the first child of healthy consanguineous parents, without pathological familiar history. Delivery was preterm via caesarean section because of foetal distress. Her weight was 1.720 kg, height of 41.5 cm, and an occipitofrontal circumference of 29 cm. The Apgar scores immediately after delivery and at 5 minutes were 3 and 8, so she was given neonatal resuscitation. She was observed in the neonatal care unit for the first three months of life because of respiratory distress episodes. Chromosome analysis showed a normal karyotype, abdominal and ophtamological exams were unremarkable. The patient was then referred to the paediatric unit after the respiratory distress episodes were treated. Her developmental milestones were incompletely informed: social smile and holding head at 3 months, sitting without support at 10 months, crawling at 11 months, and standing up with support and first words at 12 months of age. After the first year of life, the patient presented with failure to thrive and neurological regression was reported as she stopped grabbing toys and pointing objects and persons. At 2 years of age, she started episodes of diarrhoea and sleepiness evolving with respiratory failure at home and was taken to the emergency room for support and management. Her respiratory failure could not be resolved, so she was referred to the paediatric intensive care unit for better support. Her laboratory findings showed consecutive high levels of serum lactate (21 mg/dL when admitted to our critical time intervention programme, 19 mg/dL at day 7, 54 mg/dL at day 14, 69 mg/dL at day 19, and 30 mg/dL at day 24 after admission; the reference lactic acid level is 4.5 - 14.4 mg/dL). Metabolic investigations included tandem mass and urinary organic acid analyses, which were normal. Ophthalmological examination showed optic atrophy and echocardiography, abdominal and doppler sonography were normal. The electroencephalography suggested parieto-occipital epileptiform discharges, and brain magnetic resonance imaging (MRI) showed bilateral high intensity T2/Flair foci in the periventricular and peritrigonal white matter, internal capsule of the posterior limb and thalamus periphery; additionally, hyperintensities were observed in the cerebral peduncle, midbrain tegmentum, dorsal pons, and medulla oblongata, with a few areas of restricted diffusion, which is typical for mitochondrial disorders (Figure 1).

**Figure 1:**
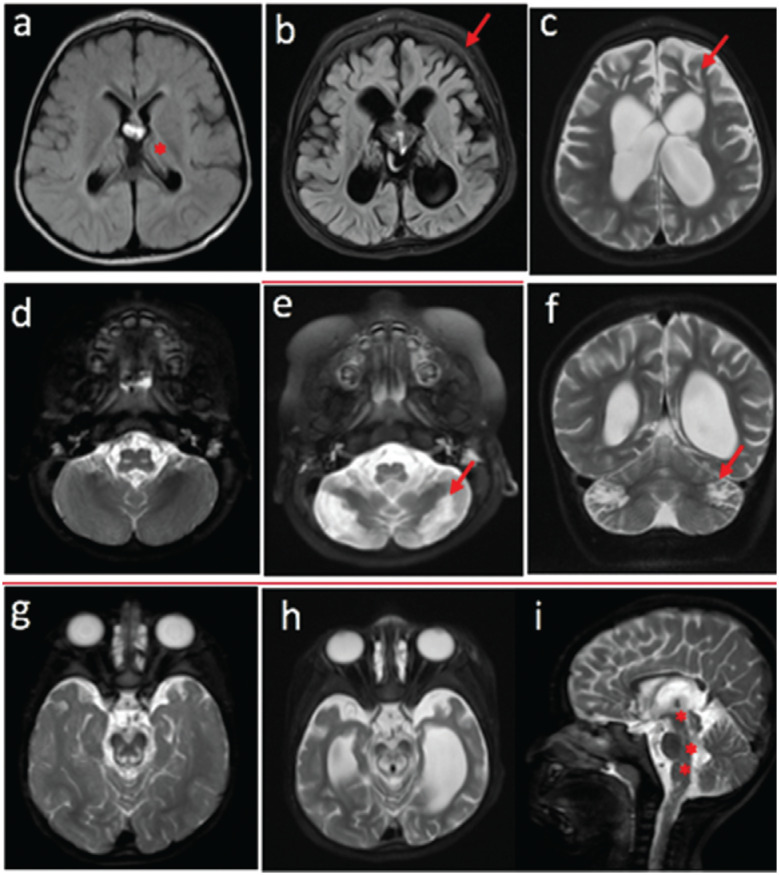
Brain magnetic resonance imaging (MRI). Axial flair sequence MRI at 2 years (a) and 2 years and 9 months (b): A symmetrical and bilateral high-intensity signal is observed the periventricular and peritrigonal white matter in addition to the posterior arm of the internal capsule (red asterisk) and periphery of the thalamus; the accentuation of the sulci and encephalic cisterns results in diffuse encephalic reduction and compensatory ectasia of the supra and infratentorial ventricular system; the diploe is thickened (red arrow), a finding not observed before, which suggests progressive cerebral atrophy (b). Axial T2-weighted imaging at 2 years and 9 months (c) demonstrating symmetric focus with the same CSF sign in the two images in bilateral frontal white matter, suggesting enlargement of Virchow-Robin spaces, although lesion of cystic degeneration or encephalomalacia cannot be excluded. Axial T2-weighted imaging at ages 2 years (d) 2 years and 9 months (e), and coronal T2-weighted sequences at 2 years and 9 months of age (f): note the symmetrical high-intensity signal in the cerebellar hemispheres. Axial T2-weighted sequence at 2 years of age (g) and axial and sagittal T2-weighted imaging at age 2 years and 9 months (h,i): note the bilateral and symmetrical high-intensity signal of the substantia nigra of the cerebellar peduncle, mesencephalic segment, posterior region of the bridge, superior cerebellar peduncle and medulla oblongata.

Empirical mitochondrial vitamin cocktail therapy, including thiamine, riboflavin, coenzyme Q10, biotin and L-carnitine, was initiated without clinically detectable improvement. Her treatment outcome was poor, with oxygen dependence, spastic tetraplegy, and severe developmental delay.

Clinical exome sequencing was then performed. Informed consent was obtained from the patient's parents, according to the ethical principles of the Declaration of Helsinki. Genomic DNA was isolated from peripheral blood samples (QIAamp DNA Blood Mini Kit; Qiagen, Hilden, Germany), and was quantified by using the Qubit dsDNA HS Assay kit for the Qubit 2.0 Fluorometer (Life Technologies, Carlsbad, CA). We used the TruSightᵃ One Sequencing Panel (Illumina, Inc., San Diego, CA, USA) for paired-end sequencing with a v3 600 cycles kit on a MiSeq System platform (Illumina), according to the manufacturer's guidelines. The FASTQ files were aligned to the Human Genome Reference Consortium build 37 (hg19) by using the Burroughs Wheeler Alignment algorithm as implemented in the BWA software package. After genome alignment, Variant Calling was performed by using the Genome Analysis Toolkit (GATK). Variants were annotated by using ANNOVAR, and selected read alignments were visualised by using the Integrative Genomics Viewer. All regions with a sequencing depth < 20 were considered unsuitable for analysis. Furthermore, we established a minimum threshold Phred-like quality score of 30 (base call accuracy of 99.9%). Common variants (3 1% in the general population) were discarded by comparison with the 1000 Genomes, the Exome Variant Server, and the Exome Aggregation Consortium database. Synonymous variants were filtered and only exonic and splice site variants were prioritized. Initially, we evaluated the variants in the homozygous state, because the parents of the proband were consanguineous, and prioritized genes based on phenotypic databases, such as the Online Mendelian Inheritance in Man. The variant identified as pathogenic was confirmed by Sanger sequencing.

We identified a novel homozygous 14 bp frameshift deletion in exon 2 of the *C12orf65* gene: c.207_220del; p.Pro70Asnfs*28 (NM_152269), which was validated by Sanger sequencing, the sequence, being aligned with CRISP-ID (Figure S1). This variant is not present in population control databases (Exome Aggregation Consortium, 1000 genomes and Exome Variant Server). The truncated protein is predicted to have 96 aa as opposed to the original length of 166 aa, lacking the GCQ domain (residues 57-121) and the coiled-coil region (residues 127-160) (UniProt databank); this likely results in loss of function. Based on these features and according to the American College of Medical Genetics and Genomics guidelines, this variant is considered to be pathogenic (Richards *et al.*, 2015).

To our knowledge, 27 patients were described with pathogenic variants in the *C12orf65* gene with different clinical diagnoses (Table S1). The phenotype can vary from spastic paraparesis - type 55 - to Leigh syndrome. It has been thought that the main clinical features caused by *C12orf65* variants correspond to a triad of optic atrophy, peripheral neuropathy, and spastic paraparesis, associated or not with Leigh syndrome. The information available from 27 patients showed that 92.3% presented with optic atrophy, 80% with peripheral neuropathy, 60% with spasticity, 52.1% with cognitive impairment, 42.8% with nystagmus, 38.4% with ophthalmoplegia, and 20% with bulbar disfunction. Our patient had optic atrophy, bulbar dysfunction (manifested as dysphagia and respiratory failure), and neurodevelopmental delay. We could not evaluate nystagmus and ophthalmoplegia, and spasticity was not present at the time of examination; the MRI findings were typical for Leigh syndrome. Our patient could be included in the COXPD7 severe phenotype, as she had a dramatically deteriorating course as described by Antonicka *et al.* (2010). This phenotype consists of significant cognitive and motor impairment and results in wheelchair dependence and typically abnormal Leigh syndrome brain MRI. Moreover, our patient showed a more severe phenotype when compared to patients described previously. The severity of this phenotype can be explained by the variant found, which affects an important functional protein domain (Figure 2).

**Figure 2:**
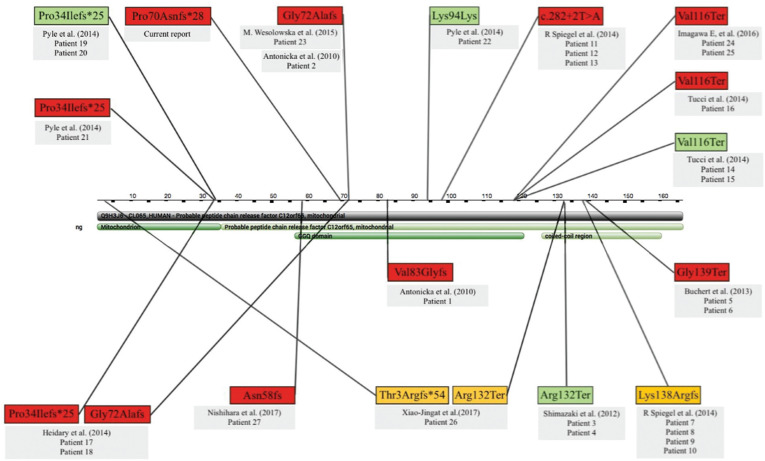
Protein structure and distribution of variants in the *C12orf65* gene. The yellow boxes represent the variants described in patients with mild phenotypes, the red boxes the variants in patients with severe phenotype, and the green boxes the variants in patients in whom it was not possible to classify disease severity based on the available clinical information. If just one variant is associated with one patient it was found in the homozygous state; two variants linked to one patient indicates compound heterozygosis. Patients are numbered as in Table S1.

Regarding the molecular status, the 27 described patients had 11 different pathogenic variants in the *C12orf65* gene (Table S1). These variants were predominantly nonsense, splicing site and frameshift, and considered to be null variants. Only one of them, c.282G > A (p.Lys94Lys), was synonymous, but it was predicted to create a loss of splicing site with retention of intron 2, thus acting as a frameshift variant. The variant found in our patient was also a null variant, and we suggest that truncated *C12orf65* proteins were generated after the deletion of 14 base pairs, which reinforces the idea that a loss-of-function mechanism is a fundamental part of the pathogenesis.

We classified the phenotype of the reviewed patients as severe when there was bulbar dysfunction, or typical MRI findings of Leigh syndrome, or intellectual disability/neurodevelopmental delay, or absence of ambulation. The variants linked to severe phenotypes were more likely to affect the GCQ domain of the protein, although they could impact other protein domains (Figure 2).

The reviewing of clinical features and variants of 27 already described patients and the patient herein shows that the phenotype associated with *C12orf65* variants is heterogeneous and that variants impacting the GGQ protein domain tend to be associated with more severe phenotypes. This is in agreement with Spiegel *et al.* (2014), who concluded that a clear genotype-phenotype correlation is anticipated, with deleterious mutations that disrupt the GGQ-containing domain being expected to result in a more severe phenotype, whereas downstream C-terminal alterations might result in a more favourable phenotype, typically lacking cognitive impairment.

Our review also showed that the median age for the onset of symptoms was 4 years and the median age for diagnosis was 17 years, so there was a 13-year diagnostic delay. Our patient presented the first symptoms immediately after birth, and the diagnosis was made two years later. A diagnostic delay may occur because of a variable phenotypic expression, despite the same aetiology (pathogenic variants in the *C12orf65* gene), the rarity of the disease, the lack of knowledge about its existence and because of difficult access to molecular confirmation. Compared with literature data, our diagnostic delay was shorter, probably due to our patient having classical MRI signals of Leigh syndrome and elevated serum biomarkers (e.g., lactate), as well as a severe phenotype that contributed to active search for the aetiology. The prompt access to exome sequencing allowed a molecular diagnosis of the disorder.

In conclusion, the present study allowed the identification of a *C12orf65* gene variant associated with a severe phenotype of Leigh syndrome caused by COXPD7 and reinforces the importance of the GGQ protein domain for the conserved peptidyl-hydrolase function. Furthermore, this study highlights that the mechanism of loss of function has an important role in the pathogenesis of the disease. Additional reports could help to better understand genotype-phenotype correlations.

## Supplementary material

The following online material is available for this article:

Table S1 - Clinical and molecular features of 27 patients with *C12orf65* pathogenic variants from the literature and the present case.

Figure S1 - Alignment of sequence in CRISP-ID V1.1a.
