# Supplementary material for: Leigh syndrome in a patient with a novel C12orf65 pathogenic variant: case report and literature review
Source: Genet Mol Biol. 2020 May 29;43(2):e20180271. doi: 10.1590/1678-4685-GMB-2018-0271 (PMC7263430; doi:10.1590/1678-4685-GMB-2018-0271)

Genetics and Molecular Biology 43, 2, (2020)

Supplementary Material to "Leigh syndrome in a patient with a novel *C12orf65* pathogenic variant: case report and literature review"

Figure S1 - Alignment of sequence in CRISP-ID V1.1a. (http://crispid.gbiomed.kuleuven.be/). The reference sequence (NG_027517.1) was inserted and aligned with forward and reverse sequencing files (ab1), showing the *C12orf65* deletion.

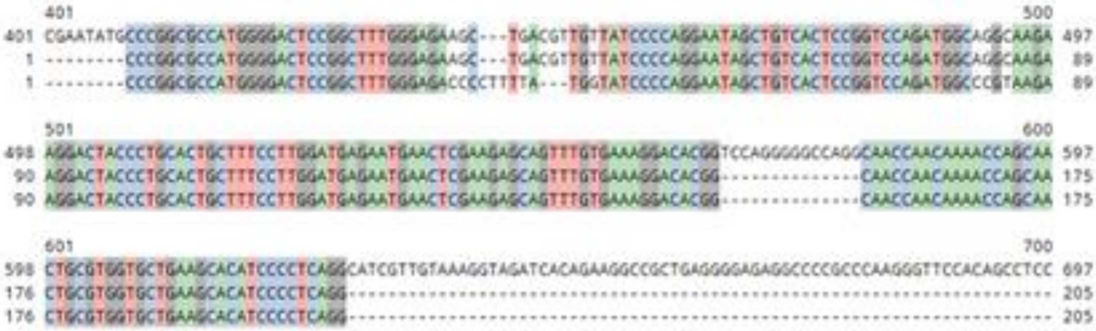

Supplement: Supplementary file 1 [file 1415-4757-GMB-43-2-e20180271-s2.pdf]
