# Supplementary material for: Leigh syndrome in a patient with a novel C12orf65 pathogenic variant: case report and literature review
Source: Genet Mol Biol. 2020 May 29;43(2):e20180271. doi: 10.1590/1678-4685-GMB-2018-0271 (PMC7263430; doi:10.1590/1678-4685-GMB-2018-0271)
Supplement: Supplementary file 2 [file 1415-4757-GMB-43-2-e20180271-s1.pdf]

Genetics and Molecular Biology 43, 2, (2020)

Supplementary Material to "Leigh syndrome in a patient with a novel *C12orf65* pathogenic variant: case report and literature review"

Table S1 - Clinical and molecular features of 27 patients with *C12orf65* pathogenic variants from the literature and the present case.

References	Patient number	Gender	Age (Onset of Symptoms)	Age (Diagnosis)	Optical atrophy	Nystagmus	Ophtalmoplegia	Peripheral Neurophaty	Spasticity	Bulbar dysfunction	Gait Absence	Cognitive Impairment	C12orf65 variant	Leigh BMR findings	Disability
Antonicka *et al.* (2010)	1	Female	1 year	Pos-mortem	+	+	+	NR	-	+	+	+	c.248delT, p.Val83Glyfs*13 (homozygous)	+	Severe
Antonicka *et al.* (2010)	2	Male	1,5 years	17 years	+	+	+	+	-	+	+	+	c.210delA, Gly72Alafs (homozygous)	-	Severe
Shimazaki *et al.* (2012)	3	Male	7 years	32 years	+	-	-	+	+	-	-	-	c.394C>T, p.Arg132Ter (homozygous)	NR	NR
Shimazaki *et al.* (2012)	4	Male	7 years	42 years	+	-	-	+	+	-	-	-	c.394C>T, p.Arg132Ter (homozygous)	NR	NR
R Buchert *et al.* (2013)	5	Female	NA	27 years	-	NR	+	NR	+	-	+	+	c.415C>T, p.Gly139Ter (homozygous)	NR	Severe
R Buchert *et al.* (2013)	6	Male	NA	24 years	-	NR	+	NR	-	-	NR	+	c.415C>T, p.Gly139Ter (homozygous)	-	Severe
R Spiegel *et al.* (2014)	7	Male	4 years	20 year	+	-	-	-	+	-	-	-	c.413_417 delAACAA, p.Lys138Argfs*17 (homozygous)	-	Mild
R Spiegel *et al.* (2014)	8	Male	5 year	7 years	+	-	-	NR	-	-	-	-	c.413_417 delAACAA, p.Lys138Argfs*17 (homozygous)	-	Mild
R Spiegel *et al.* (2014)	9	Male	3,5 years	23 years	+	-	-	+	+	-	-	-	c.413_417 delAACAA, p.Lys138Argfs*17 (homozygous)	-	Mild
R Spiegel *et al.* (2014)	10	Female	6 years	18 years	+	-	-	+	+	-	-	-	c.413_417 delAACAA,	-	Mild

References	Patient number	Gender	Age (Onset of Symptoms)	Age (Diagnosis)	Optical atrophy	Nystagmus	Ophtalmoplegia	Peripheral Neurophaty	Spasticity	Bulbar dysfunction	Gait Absence	Cognitive Impairment	C12orf65 variant	Leigh BMR findings	Disability
													p.Lys138Argfs*17 (homozygous)		
R Spiegel *et al.* (2014)	11	Female	1 year	17 years	+	+	-	+	+	-	-	+	g.21043T>A (c.282 + 2 T>A) (homozygous)	-	Severe
R Spiegel *et al.* (2014)	12	Male	1 year	12 years	+	+	-	+	+	-	-	+	g.21043T>A (c.282 + 2 T>A) (homozygous)	+	Severe
R Spiegel *et al.* (2014)	13	Female	1 year	8 years	+	+	-	+	+	-	-	+	g.21043T>A (c.282 + 2 T>A) (homozygous)	+	Severe
Tucci A *et al.* (2014)	14	Male	8 years	34 years	+	NR	-	+	NR	-	NR	-	c.346delG, p.Val116Ter (homozygous)	NR	NR
Tucci A *et al.*(2014)	15	Male	NR	NR	NR	NR	NR	NR	NR	NR	NR	NR	NR[†] (probably c.346delG; p.V116Ter)	NR	NR
Tucci A *et al.* (2014)	16	Female	NR	35 years	+	NR	-	+	+	-	-	+	c.346delG, p.Val116Ter (homozygous)	NR	Severe
Heidary *et al.* (2014)	17	Male	9 months	7 years	+	+	-	-	-	-	-	-	c.96_99dupATC, p.Pro34IlefsX25/ c.210delA, p.Gly72Alafs*13	+	Severe
Heidary *et al.* (2014)	18	Female	NR	4 years	+	-	+	-	-	+	NR	NR	c.96_99dupATC, p.Pro34IlefsX25/ c.210delA, p.Gly72Alafs*13	+	Severe
Pyle *et al.* (2014)	19	Male	5 years	13 years	+	+	+	+	+	NR	-	NR	c.96_99dupATCC, p.Pro34Ilefs*25 (homozygous)	-	NR
Pyle *et al.* (2014)	20	Female	6 years	7 years	+	-	+	NR	+	NR	-	NR	c.96_99dupATCC, p.Pro34Ilefs*25 (homozygous)	-	NR
Pyle *et al.* (2014)	21	Male	5 years	22 years	+	-	-	-	+	-	-	+	c.96_99dupATCC, p.Pro34Ilefs*25 (homozygous)	+	Severe
Pyle *et al.* (2014)	22	Male	1 year	16 years	+	+	-	-	-	-	-	NR	c.282G>A, p.Lys94Lys (homozygous)	NR	NR
M. Wesolowska *et al.* (2015)	23	Male	2 years	45 years	+	-	+	NR	-	+	-	-	c.210delA, p.Gly72Alafs*13 (homozygous)	+	Severe

References	Patient number	Gender	Age (Onset of Symptoms)	Age (Diagnosis)	Optical atrophy	Nystagmus	Ophtalmoplegia	Peripheral Neurophaty	Spasticity	Bulbar dysfunction	Gait Absence	Cognitive Impairment	C12orf65 variant	Leigh BMR findings	Disability
Imagawa E, *et al*. (2016)	24	Female	6 months	11 years	+	+	+	-	+	-	-	+	c.346delG, p.Val116Ter (homozygous)	+	Severe
Imagawa E, *et al*. (2016)	25	Female	6 months	11 years	+	NR	+	-	+	-	NR	+	c.346delG, p.Val116Ter (homozygous)	+	Severe
Xiao-Jing *et al*. (2017)	26	Female	3 years	8 years	+	-	-	+	-	-	-	-	c.394C>T, p.Arg132Ter/ c.6_7delCA, p.Thr3Argfs*54	-	Mild
Nishihara *et al*. (2017)	27	Female	NR	NR	+	-	-	+	-	+	-	+	c.171_172del GA, p.Arg58fs (homozygous)	+	Severe
Current Report	28	Female	1st day of life	2 years	+	NR	NR	NR	-	+	NR	+	c.207_220del , p.Pro70Asnfs*28 (homozygous)	+	Severe

Legend

NR: not reported

✢Probably c.346delG (p.V116X), considering he is from the same pedigree as 14 and 16
